# Clinicopathologic significance of the CXCL1-CXCR2 axis in the tumor microenvironment of gastric carcinoma

**DOI:** 10.1371/journal.pone.0178635

**Published:** 2017-06-02

**Authors:** Hiroaki Kasashima, Masakazu Yashiro, Hirohisa Nakamae, Go Masuda, Haruhito Kinoshita, Tamami Morisaki, Tatsunari Fukuoka, Tsuyoshi Hasegawa, Takahiko Nakane, Masayuki Hino, Kosei Hirakawa, Masaichi Ohira

**Affiliations:** 1 Department of Surgical Oncology, Osaka City University Graduate School of Medicine, Osaka, Japan; 2 Molecular Oncology and Therapeutics, Osaka City University Graduate School of Medicine, Osaka, Japan; 3 Department of Hematology, Osaka City University Graduate School of Medicine, Osaka, Japan; University of South Alabama Mitchell Cancer Institute, UNITED STATES

## Abstract

**Purpose:**

It was reported that the chemokine (C-X-C motif) ligand 1 (CXCL1) from cancer cells stimulated the recruitment of bone marrow-derived mesenchymal cells (BM-MCs) into tumor stroma via chemokine (C-X-C motif) receptor 2 (CXCR2) signaling. We conducted this retrospective study to determine the clinicopathologic significance of the CXCL1-CXCR2 axis in human gastric cancer.

**Methods:**

The correlations between the clinicopathological features of 270 primary gastric carcinomas and CXCL1 in cancer cells and CXCR2 in stromal cells were analyzed in immunohistochemical studies. The effect of gastric cancer cells on the expression of CXCR2 in BM-MCs was examined using diffuse-type gastric cancer cell lines *in vitro*.

**Results:**

The expression of CXCL1 in cancer cells was correlated with T invasion (T2–T4), lymph node metastasis, lymphatic invasion, venous invasion, peritoneal cytology, peritoneal metastasis and CXCR2 expression in stromal cells. The expression of CXCR2 in stromal cells was correlated with macroscopic type-4 cancers, histological type, T invasion (T2–T4), lymph node metastasis, lymphatic invasion, infiltration, peritoneal cytology, peritoneal metastasis and CD271 expression in stromal cells. The overall survival of patients with CXCL1 and CXCR2-positive cancer was poorer than that of the patients with negative cancer. Both CXCL1 expression in cancer cells and CXCR2 expression in stromal cells were independent prognostic factors for gastric cancer patients.

**Conclusion:**

The expressions of CXCL1 in cancer cells and CXCR2 in stromal cells are useful prognostic factors for gastric cancer patients.

## Introduction

Cancer progression and metastasis are controlled by the tumor microenvironment and do not depend solely on cancer cell-autonomous defects [1, 2]. A major component of the tumor stroma is fibroblasts, and activated fibroblasts play a critical role in the regulation of solid tumor progression [3]. The interaction between cancer cells and cancer-associated fibroblasts (CAFs) has been suggested to be important for the progression of some types of cancer [4–6], and we demonstrated that some amount of CAFs in the stroma of gastric cancer originated from bone marrow-derived mesenchymal cells (BM-MCs) [7]. We also observed that the stromal expression of CD271, which is one of the markers of BM-MCs, was a useful prognostic factor for gastric cancer patients [7]. Another of our studies demonstrated that chemokine (C-X-C motif) ligand 1 (CXCL1), which is produced from diffuse-type gastric cancer (DGC) cells, is closely associated with the recruitment of BM-MCs into tumor stroma via chemokine (C-X-C motif) receptor 2 (CXCR2) of BM-MCs [8].

Several research groups reported that CXCL1 [9, 10] and CXCR2 [11, 12] were poor prognostic factors in studies using human samples of several types of cancers including gastric cancer, colorectal cancer, and pancreatic cancer. Ijichi et al. also reported that CXCL1/CXCR2 signaling regulated tumor-stromal interactions in pancreas cancer [13]. CXCL1 hypersecretion from colorectal cancers' epithelia and myofibroblasts was also related with poor prognosis [9]. However, we have found no report of the clinical significance of the CXCL1-CXCR2 axis within cancer cells and stromal cells in gastric cancer. We thus extended the scope of our research to determine the significance of the CXCL1-CXCR2 axis in the tumor microenvironment of gastric cancer.

## Materials and methods

### Clinical materials

Human gastric cancer tissues were obtained from 270 patients who had undergone resection of primary gastric cancer at our institution. The pathological diagnoses and classifications were made according to the Japanese classification of gastric carcinoma (14^th^ edition) [14]. This study was approved by the Osaka City University Ethics Committee (approval number 924). Written informed consent from the donor was obtained for use of this sample in research.

### Immunohistochemical determination

The immunohistochemical determination of CXCL1 and CXCR2 were examined as the manufacturer’s instructions. Shortly, slides were deparaffinized and heated for 10 min at 105°C in an autoclave with Target Retrieval Solution (Dako, Carpinteria, CA). After blocking endogenous peroxidase activity, the samples were incubated with anti-CXCR2 antibody (1:50, R&D Systems, Minneapolis, MN) or anti-CXCL1 antibody (1:100, Abcam, Cambridge, UK) antibody for 1 h at room temperature. The samples were incubated with biotinylated goat anti-rabbit IgG for 10 min. The samples were treated with streptavidin-peroxidase reagent, and counterstaining with Mayer’s hematoxylin. CXCL1 and CXCR2 expressions were evaluated at the invading tumor front by intensity of staining and percentage of stained cancer cells and stromal cells, respectively. CXCL1 and CXCR2 expression was evaluated by intensity of staining and percentage of stained cancer cells and stromal cells, respectively: intensity was given scores 0–3 (0 = no, 1 = weak, 2 = moderate, 3 = intense), and the percentage of positive cells was given scores 0–4 (0 = 0%, 1 = 1–20%, 2 = 21–60%, 3 = 61–80%, 4 = 81–100%). The two scores were added to obtain the final result of 0–7. Expressions were considered positive when scores were 4 or more and negative when scores were 3 or less. Two double-blinded independent observers who were unaware of clinical data and outcome evaluated, and when a discrepant evaluation between the two independent observers was found, the result was rechecked and discussed.

### Cell lines

Two DGC cell lines, OCUM-2M[15] and OCUM-2MD3[16], were used. The culture medium consisted of Dulbecco’s modified Eagle medium (DMEM; Nikken, Kyoto, Japan) with the addition of 10% fetal bovine serum (FBS; Nichirei, Tokyo, Japan), 100 IU/ml penicillin (Wako, Osaka, Japan), 100 mg/ml streptomycin (Wako), and 0.5mM sodium pyruvate (Wako). Cells were cultured at 37°C in 21% O_2_. Human bone marrow derived mesenchymal cells (BM-MCs) were obtained from bone marrow of the iliac crest of donors who were underwent bone marrow examination because of hematological disorders, and was proven to be normal tissues and cultured in a dish with MesenCult™-XF Medium (STEMCELL Technologies, Grenoble, France) as previously reported[7]. Adherent cells on dish were collected and transferred to another culture dish every 7 days in fresh medium. This study was approved by the Osaka City University ethics committee, and informed consent was obtained from patients prior to study. In culture medium, BM-MCs formed a monolayer of adherent cells and looked like long spindle-shaped fibroblastic cells.

### Preparation of conditioned medium

We collected conditioned medium (CM) from OCUM-2M and OCUM-2MD3. To obtain these CM, cells were washed with PBS three times and incubated for 3 days in 1.5 ml DMEM without FBS. The supernatant was stored as CM at -80°C until use. All experiments were performed in medium contain 2% FCS. As a control, DMEM was used instead of CM.

### Quantitative real-time reverse transcription-polymerase chain reaction (RT-PCR)

Real-time RT-PCR was analyzed using the ABI Prism 7000 (Applied Biosystems, Foster City, CA), as previously described[7]. Total cellular RNA was extracted from cells with Trizol (Life Technologies). Relevant cDNA was amplified by PCR with Taq DNA polymerase (Nippon Gene, Tokyo, Japan) with a thermal cycler. Primer sets for *CXCR2* (assay ID: Hs01891184) were from Applied Biosystems. The primer set for *GAPDH*, as an internal control, (accession numbers NM_002046; probe, 5’-CCCCTGCAAATGAGCCCCAGCCTTC-3’; forward, 5’- CCATCTTCCAGGAGCGAGATC-3’; reverse, 5’-GGCAGAGATGATGACCCTTTTG-3’) was purchased from Sigma-Aldrich (St. Louis, MO). RT-PCR was performed at 95℃ for 15 seconds and then 60℃ for 60 seconds for 40 cycles.

### Statistical analysis

The chi-square test or Fisher’s exact was used to calculate the significance of differences between covariates. Survival durations were analyzed by the Kaplan-Meier method and calculated with the log-rank test to compare cumulative survival durations among each patient groups. Additionally, the Cox proportional hazards model was used to compute univariate hazards ratios for the study parameters. Generally, p<0.05 was defined as statistically significant. The SPSS software program (SPSS Japan, Tokyo, Japan) was used for the analyses. *In vitro*, Data are expressed as mean ± SD and significant difference was analyzed using the unpaired Student’s *t*-test.

## Results

### Relationship between clinicopathological features and CXCL1 expression in cancer cells or CXCR2 expression in stromal cells

**Fig 1:**
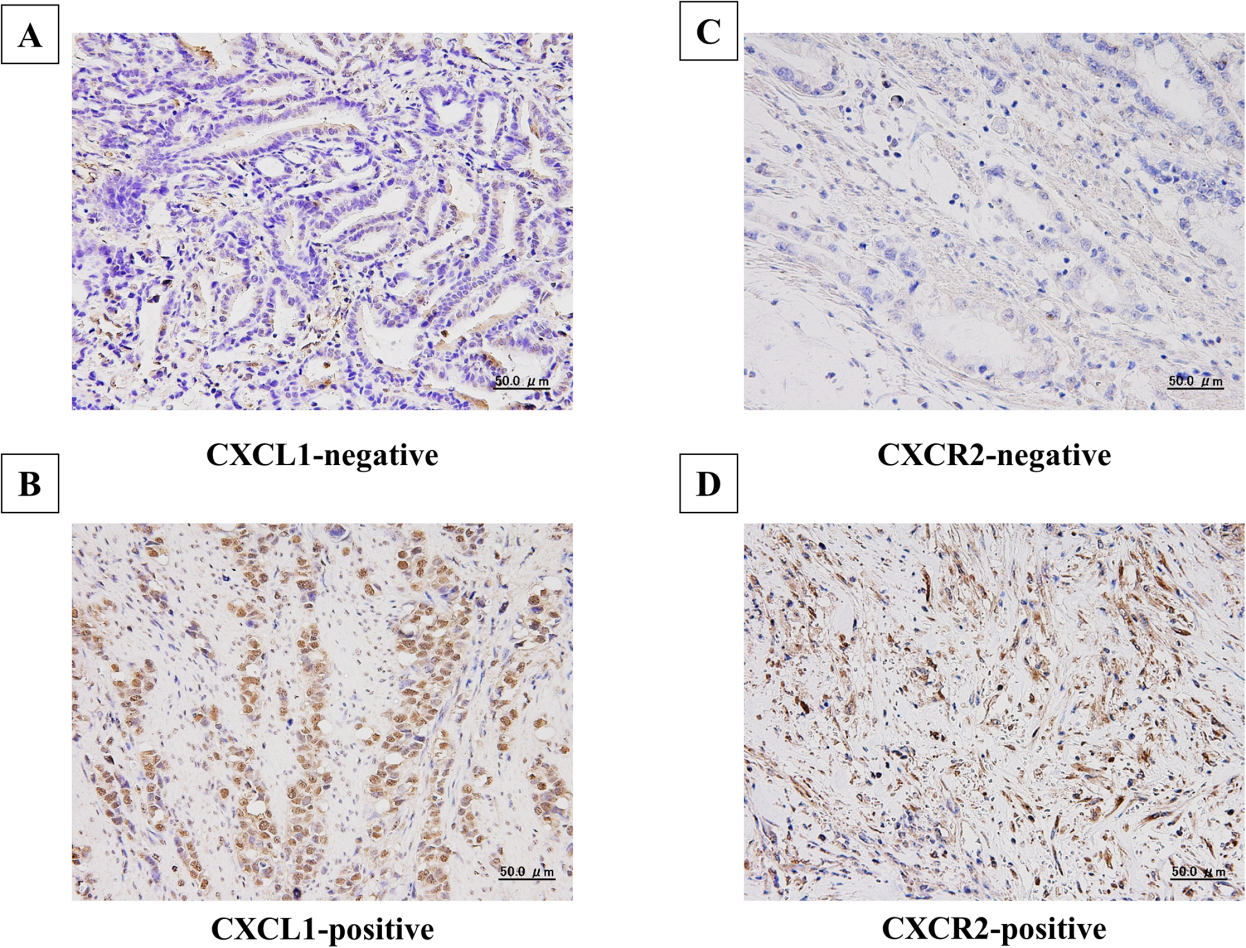
Representative picture of CXCL1 expression and CXCR2 expression in gastric cancer tissue. CXCL1 was expressed in cytoplasm in gastric cancer cells **(B)**. CXCR2 was expressed on the membrane in stromal cells **(D)**.

CXCL1 was expressed in the cytoplasm of gastric cancer cells, and CXCR2 was expressed on the cell membrane and in the cytoplasm of fibroblasts (Fig 1). Among the 270 gastric cancers examined, 144 cases (53%) were positive for CXCL1 expression in cancer cells, and 113 cases (42%) were positive for CXCR2 expression in stromal cells. In addition, 83 cases (33%) were negative for both types of expression and 74 cases (29%) were positive for both types of expression. Table 1 shows the correlations between the clinicopathological features and CXCL1 expression in cancer cells and CXCR2 expression in stromal cells. CXCL1 expression in cancer cells was significantly correlated significantly with T invasion (T2–T4), lymph node metastasis, lymphatic invasion, venous invasion, peritoneal cytology, peritoneal metastasis, and CXCR2 expression in stromal cells. CXCR2 expression in stromal cells was significantly correlated with macroscopic type-4 cancers, histological type, T invasion (T2–T4), lymph node metastasis, lymphatic invasion, infiltration, peritoneal cytology, peritoneal metastasis and CD271 expression in stromal cells.

**Table 1:** Correlation between the expression of CXCL1 in cancer cells and that of CXCR2 in stromal cells and clinicopathologic features in 270 patients with gastric carcinoma.

Clinicopathologic features		CXCL1 expression in cancer cells		*p*-value	CXCR2 expression in stromal cells		*p*-value
		Positive (n = 144)	Negative (n = 120)		Positive (n = 113)	Negative (n = 157)	
Age	<70	75(49%)	77(51%)	0.061	65(42%)	90(58%)	1.000
	≥70	69(62%)	43(38%)		48(42%)	67(58%)	
Gender	female	75(50%)	75(50%)	0.105	58(38%)	96(62%)	0.135
	male	69(61%)	45(39%)		55(47%)	61(53%)	
Macroscopic type	Other type	126(53%)	114(47%)	0.051	94(39%)	150(61%)	0.001
	Type-4	18(75%)	6(25%)		19(73%)	7(27%)	
Histological type	Intestinal-type	72(57%)	54(43%)	0.459	37(30%)	86(70%)	<0.001
	Diffuse-type	72(52%)	66(48%)		76(52%)	71(48%)	
T invasion	T1	38(30%)	88(70%)	<0.001	31(24%)	96(76%)	<0.001
	T2, T3, T4	106(77%)	32(23%)		82(57%)	61(43%)	
Lymph node metastasis	Negative	54(36%)	97(64%)	<0.001	41(27%)	111(73%)	<0.001
	Positive	90(80%)	23(20%)		72(63%)	43(37%)	
Lymphatic invasion	Negative	54(36%)	97(64%)	<0.001	41(27%)	111(73%)	<0.001
	Positive	90(80%)	23(20%)		72(63%)	43(37%)	
Venous invasion	Negative	90(44%)	115(56%)	<0.001	83(39%)	129(61%)	0.099
	Positive	54(92%)	5(8%)		30(52%)	28(48%)	
Infiltration^*^	a, b	105 (56%)	81(44%)	0.883	62(34%)	120(66%)	<0.001
	c	36(58%)	26(42%)		49(67%)	24(33%)	
Hepatic metastasis	Negative	138(54%)	118(46%)	0.298	108(41%)	154(59%)	0.285
	Positive	6(75%)	2(25%)		5(63%)	3(37%)	
Peritoneal cytology	Negative	119(54%)	102(46%)	0.020	93(42%)	129(58%)	0.005
	Positive	17(81%)	4(19%)		18(72%)	7(28%)	
Peritoneal metastasis	Negative	131(53%)	118(47%)	0.014	102(40%)	152(60%)	0.035
	Positive	13(87%)	2(13%)		11(69%)	5(31%)	
CD271 expression in stromal cells	Negative	66(58%)	47(42%)	0.379	33(30%)	76(70%)	0.001
	Positive	75(53%)	67(47%)		78(51%)	76(49%)	
CXCR2 expression in stromal cells	Negative	64(44%)	83(56%)	<0.001			
	Positive	74(70%)	32(30%)				

* INF; Infiltration pattern of tumor. The predominant pattern of infiltrating growth into the surrounding tissue is classified as follows; INF a: The tumor shows expanding growth and a distinct border with the surrounding tissue. INF b: This category is between INF a and INF b. INF c: The tumor shows infiltrating growth and an indistinct border with the surrounding tissue.

### Patients' survival

**Fig 2:**
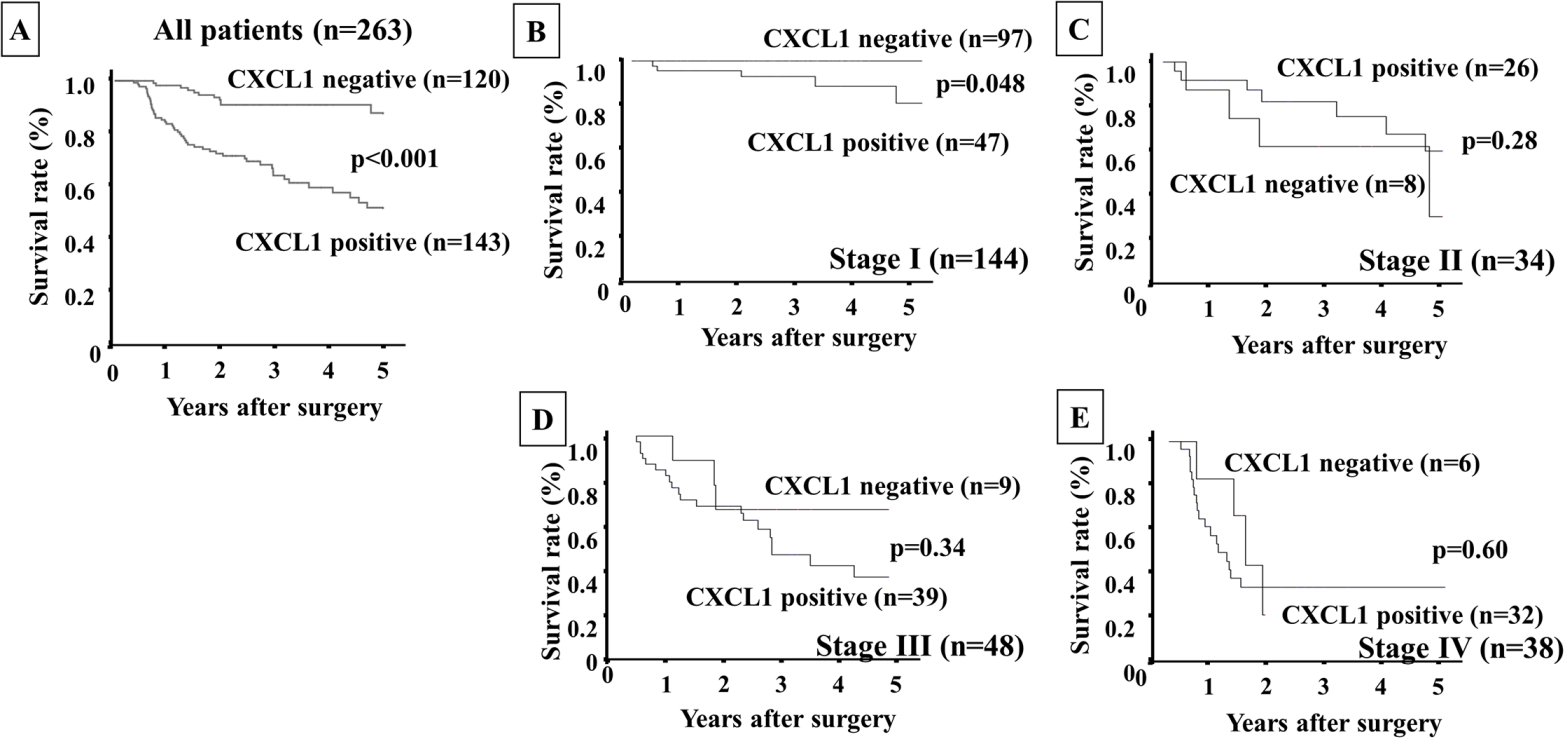
Overall survival of gastric cancer patients based on CXCL1 expression in gastric cancer cells. **A:** The Kaplan-Meier survival curve indicated that the overall survival of all patients with CXCL1-positive expression in cancer cells was significantly worse than that of the patients with CXCL1-negative expression (*p*<0.001). **B–E:** The Kaplan-Meier survival curve for each stage. Among only the patients at stage I, the patients with CXCL1-positive expression had poorer prognoses than those without CXCL1 expression.

**Fig 3:**
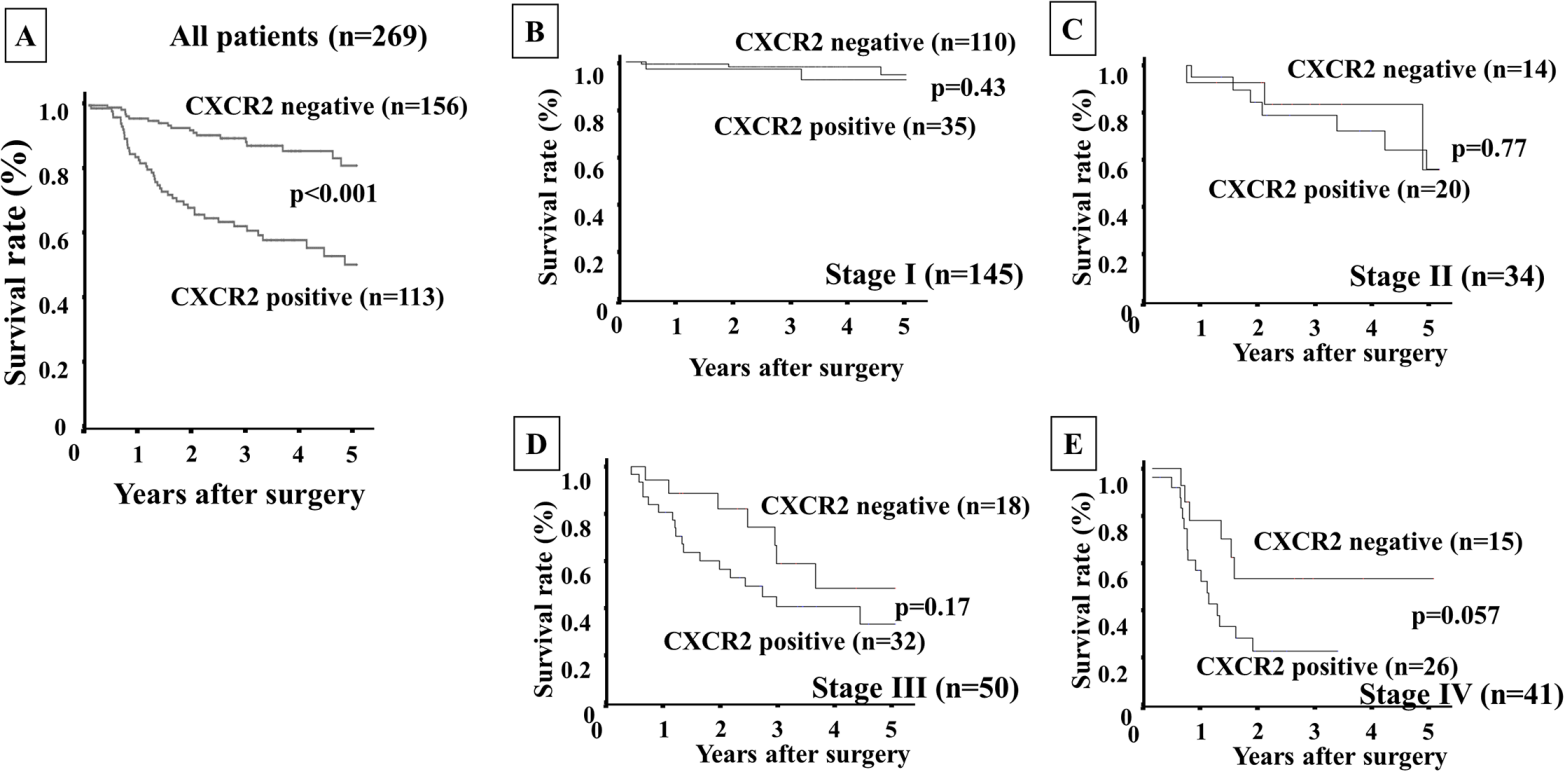
Overall survival of gastric cancer patients based on CXCR2 expression in stromal cells. **A:** The Kaplan-Meier survival curve indicated that the overall survival of all patients with CXCR2-positive expression in stromal cells was significantly worse than that of the patients with CXCR2-negative expression (*p*<0.001). **B–E:** The Kaplan-Meier survival curve for each stage. CXCR2 expression could not predict the prognosis of gastric cancer patients for each stage.

**Fig 4:**
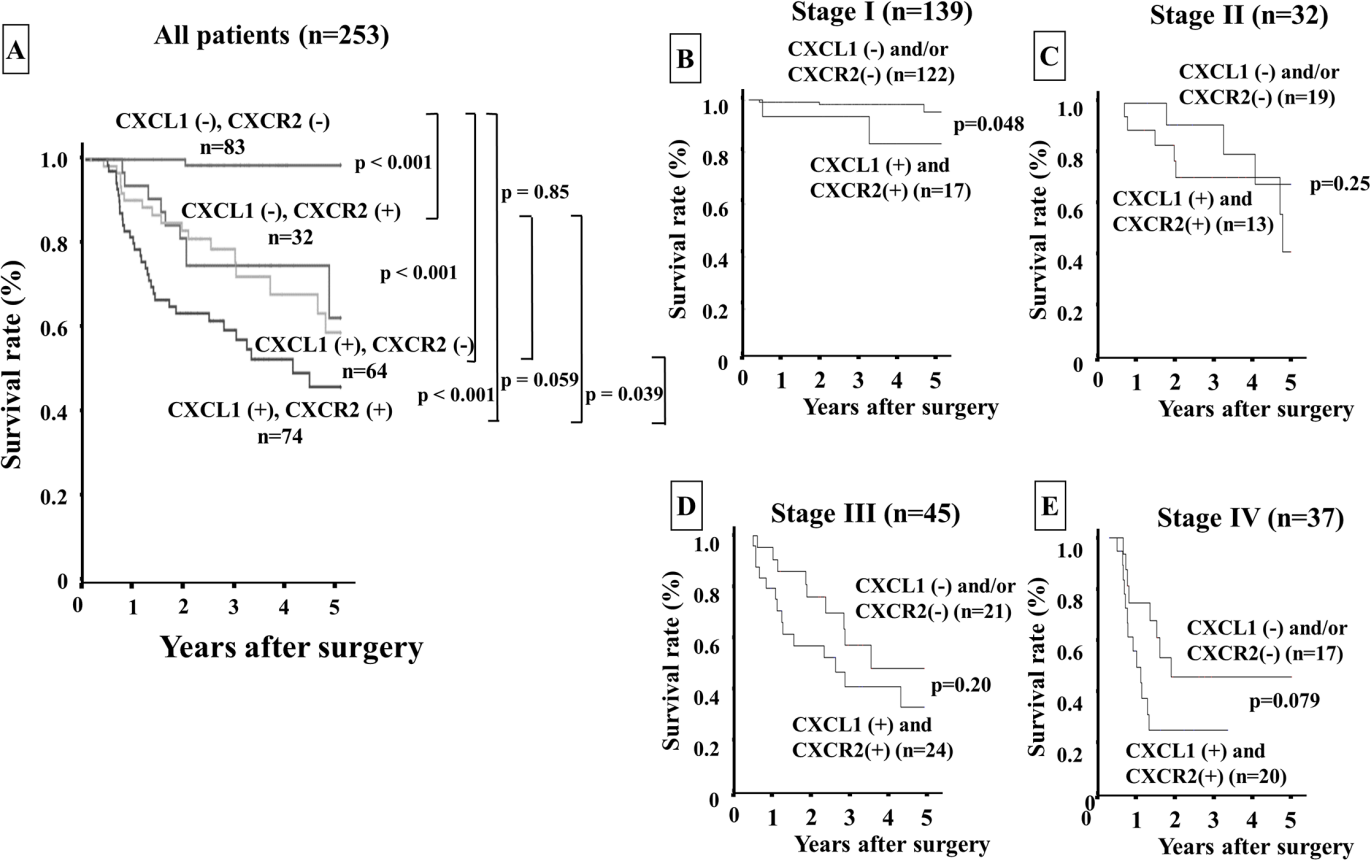
Overall survival of gastric cancer patients based on a combination of CXCL1 in cancer cells and CXCR2 in stromal cells. **A:** The Kaplan-Meier survival curve indicated that the overall survival of all patients with both CXCL1 and CXCR2 expressions was significantly worst among those of the patients with either negative or both negative expressions. **B–E:** The Kaplan-Meier survival curve for each stage. The patients with both expressions of CXCL1 in cancer cells and CXCR2 in stromal cells had significantly worse prognoses than those with either or both negative expressions in stage I.

Figs 2, 3 and 4 show the Kaplan-Meier survival curves for the gastric cancer patients in terms of CXCL1 expression in cancer cells and/or CXCR2 expression in stromal cells. The overall survival rate of all patients (n = 143) with CXCL1-positive cancer cells was significantly poorer (*p*<0.001) than that of the patients with CXCL1-negative cancer cells (n = 120) (Fig 2A). Interestingly, according to the analysis for each stage, the overall survival rate of the patients with CXCL1-positive expression at stage I (n = 47) was significantly poorer than that of the patients with CXCL1-negative expression (n = 97) (Fig 2B–2E).

Patients with CXCR2-positive stromal cells (n = 113) had significantly poorer prognoses (*p*<0.001) than those with CXCR2-negative stromal cells (n = 156) (Fig 3A). According to the analysis for each stage, there was no significant difference in prognosis based on CXCR2 expression, whereas the CXCR2 expression in stromal cells tended to show sensitive predictive power at the late stages, i.e., stage III (Fig 3D) and stage IV (Fig 3E), compared to the early stages (stage I, Fig 3B and stage II, Fig 3C). The patients who were positive for both CXCL1 in cancer cells and CXCR2 in stromal cells (n = 74) had significantly worst prognoses in comparison with the other groups (Fig 4A).

The analysis for each stage revealed that the overall survival rate of patients with both expressions was significantly poorer than that of the patient with either or both negative expressions only at stage I (Fig 4B–4E). A univariate analysis revealed that the overall survival of the patients was significantly correlated with CXCL1 expression in cancer cells, CXCR2 expression in stromal cells, both expressions (CXCL1 in cancer cells and CXCR2 in stromal cells), macroscopic type-4 cancers, diffuse-type cancers, T invasion (T2–T4), lymph node metastasis, lymphatic invasion, venous invasion, infiltration, hepatic metastasis, peritoneal metastasis, and peritoneal cytology. A multivariable logistic regression analysis revealed that CXCR2 expression in stromal cells, the expression of both CXCL1 in cancer cells and CXCR2 in stromal cells, T invasion (T2–T4), lymph node metastasis and peritoneal cytology were independent predictive parameters for the overall survival of the patients (Table 2).

**Table 2:** Univariate and multivariate Cox multiple regression analysis with respect to overall survival after surgery in 270 patients with gastric carcinoma.

	Univariate			Multivariate		
Parameter	Hazard Ratio	95% CI	*p*-value	Hazard Ratio	95% CI	*p*-value
CXCL1 Positive	4.808	2.501–9.243	<0.001	1.697	0.813–3.541	0.159
CXCR2 Positive	3.773	2.202–6.465	<0.001	1.983	1.073–3.667	0.029
CXCL1 and CXCR2 both positive	3.654	2.187–6.104	<0.001	1.795	1.035–3.114	0.037
Macroscopic Type: Type-4	5.781	5.288–10.564	<0.001	1.985	0.979–4.025	0.057
Histological type: Diffuse-type	1.978	1.305–2.479	0.008	1.413	0.725–2.754	0.309
T invasion: T2-T4	20.037	4.992–12.756	<0.001	6.233	1.764–22.026	0.004
Lymph node metastasis: Positive	10.960	5.566–12.610	<0.001	2.863	1.241–6.609	0.014
Lymphatic invasion: Positive	8.035	3.603–8.474	<0.001	0.745	0.299–1.854	0.527
Venous invasion: Positive	3.666	2.379–4.554	<0.001	1.557	0.843–2.875	0.157
Infiltraion type c	1.831	1.315–2.511	0.014	0.563	0.268–1.183	0.129
Hepatic metastasis: Positive	5.135	3.696–11.628	<0.001	1.859	0.682–5.072	0.226
Peritoneal metastasis: Positive	10.595	6.016–13.796	<0.001	1.489	0.637–3.481	0.358
Peritoneal cytology: Positive	11.030	5.250–10.541	<0.001	3.275	1.530–7.011	0.002

### *CXCR2* mRNA expression in bone marrow-derived mesenchymal cells

**Fig 5:**
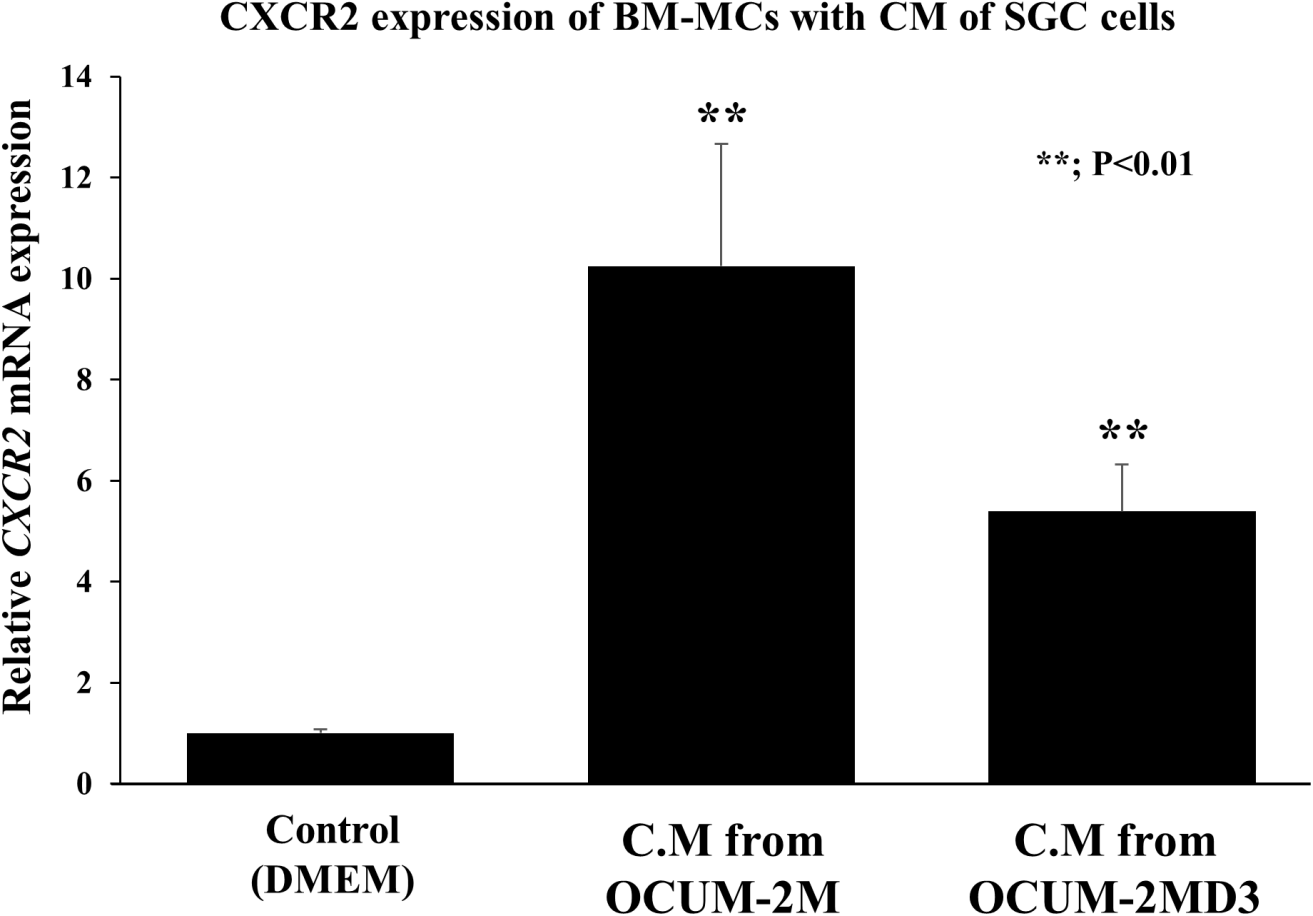
Effect of diffuse-type gastric cancer cells on CXCR2 expression of BM-MCs. Conditioned medium from diffuse-type gastric cancer cells significantly stimulated the CXCR2 expression in BM-MCs (*p*<0.01). Data are the mean and SD (error bars) of at least three experiments.

Conditioned medium (CM) from DGC cells (OCUM-2M and OCU-2MD3) significantly (*p*<0.01) upregulated the *CXCR2* mRNA expression of BM-MCs (Fig 5).

## Discussion

We previously reported that CAFs in diffuse-type gastric cancer (DGC) might originate from BM-MCs, and that CXCL1 from DGC cells stimulates the recruitment of BM-MCs into tumor stroma via CXCR2 signaling of BM-MCs *in vitro* and *in vivo* [8]. CXCL1 and CXCR2 are expressed in both gastric cancer cells and stromal cells including fibroblasts and macrophages in our study. Previously, we reported that CXCL1 secreted from gastric cancer cells might play an important role for the migration activity of BM-MCs via CXCL1/CXCR2 signaling [8]. Therefore, we focused on CXCL1 expression in gastric cancer cells and CXCR2 expression in stromal fibroblast cells in this study. In the present study, the Kaplan-Meier survival curves for the patients revealed that those with CXCL1 expression in cancer cells and those with CXCR2 expression in stromal cells had poorer survival rates than the patients without either of these expressions. Moreover, the patients with both CXCL1 and CXCR2 expressions had the worst prognoses compared to those with no expression or either expression. Of note, the patients with CXCL1 expression had poorer prognoses than those without CXCL1 expression only among the stage I patients, whereas at the later stages the patients with CXCR2 expression had a worse survival rate than those without the expression. A combination of CXCL1 and CXCR2 expression might thus be a useful prognostic indicator, especially for stage I gastric cancer patients.

The multivariate analysis revealed that CXCL1 expression in cancer cells was not an independent prognostic factor. There are several ligands of CXCR2, CXCL1-3 and CXCL5-8. These findings suggested that not only CXCL1 but also other ligands might be partially associated with CXCR2 signaling in gastric cancer. On the other hand, the protein expression of both CXCL1 in cancer cells and CXCR2 in stromal cells was significantly correlated with overall survival. These findings suggest that a combination of CXCL1 in cancer cells and CXCR2 in stromal cells may be a useful predictive prognostic factor. Hepatic metastasis and peritoneal dissemination were not independent prognostic factors. Since the number of patients with hepatic metastasis (n = 8) and peritoneal dissemination (n = 15) was small, they could not be reached to significant factors in this analysis.

CXCL1 expression in gastric cancer was shown to be associated with vascular endothelial growth factor (VEGF) and p-STAT3 expression, advanced tumor stage and poor prognosis [10]. Neutrophils express CXCR2, and its expression was correlated with poor prognosis of hepatocellular carcinoma patients [17]. CXCR2-expressing myeloid-derived suppressor cells promoted colitis-associated tumorigenesis [18]. As mentioned above, many studies reported that CXCL1 signaling in the tumor microenvironment was associated with tumor progression. Our present results might be explained as follows: CXCL1 secretion by gastric cancer cells occurs first and subsequently stromal cells with CXCR2 expression are recruited into the tumor microenvironment, which is consistent with our previous *in vitro* and *in vivo* findings [8].

Regarding the correlations between the expressions of CXCL1 and CXCR2 and clinicopathologic features, our results demonstrated that CXCL1 expression in cancer cells was associated with tumor cell invasion (T2–T4), lymph node metastasis, lymphatic invasion, venous invasion, peritoneal cytology, peritoneal metastasis and CXCR2 expression in stromal cells. These results suggested that CXCL1 secreted from gastric cancer cells might affect the expression of CXCR2 in stromal cells as well as cancer progression. Additionally, we also examined the effect of gastric cancer cells on the mRNA level of *CXCR2* in BM-MCs. Fig 5 indicated that cancer cells up-regulated the *CXCR2* expression in BM-MCs. These findings might demonstrate the close interaction between gastric cancer cells and BM-MCs.

CXCR2 expression in stromal cells was associated with CD271 expression in stromal cells in this study. We previously reported that gastric cancer patients with CD271 expression in stromal cells had poorer prognosis than those without CD271 expression [7]. CD271 is reported to be a marker of BM-MCs and represents the stemness of BM-MCs [19, 20]. These findings suggest that CXCR2 might be an indicator of BM-MCs which are recruited in the tumor microenvironment.

Our study demonstrated that the crosstalk between CXCL1 from cancer cells and CXCR2 of stromal fibroblasts cells might be associated with tumor progression. Recently, CXCL1-CXCR2 axis in tumor microenvironment becomes increasingly paid attention in several types of cancer, including gastric cancer [10], hepatocellular carcinoma[17], colorectal cancer[21], pancreatic cancer[22] and prostate cancer[23]. However, these studies reported the crosstalk between cancer cells and neutrophil[17], macrophage[21], or adipose stromal cells[23] via paracrine or autocrine manner. In contrast, our study reported the role of cancerous CXCL1 expression and CXCR2 expression of stromal fibroblasts in gastric cancer. This is the first report that demonstrates clinical significance CXCL1 expression of cancer cells and CXCR2 expression of fibroblasts. We currently reported that CXCL1 from gastric cancer cells stimulates CXCR2 signalling of BM-MCs which become myofibroblasts in tumor stroma using cell lines[8]. Our previous report and this study might suggest the significance of CXCL1/CXCR2 axis on the progression of gastric cancer via the crosstalk between CXCL1 from cancer cells and CXCR2 of stromal fibroblasts.

In conclusion, the expression of the CXCL1-CXCR2 axis in cancer cells and stromal cells are useful prognostic factors for patients with gastric cancer.
